# Cultivating intellectual community in academia: reflections from the Science and Technology Studies Food and Agriculture Network (STSFAN)

**DOI:** 10.1007/s10460-023-10439-1

**Published:** 2023-05-01

**Authors:** Karly Burch, Mascha Gugganig, Julie Guthman, Emily Reisman, Matt Comi, Samara Brock, Barkha Kagliwal, Susanne Freidberg, Patrick Baur, Cornelius Heimstädt, Sarah Ruth Sippel, Kelsey Speakman, Sarah Marquis, Lucía Argüelles, Charlotte Biltekoff, Garrett Broad, Kelly Bronson, Hilary Faxon, Xaq Frohlich, Ritwick Ghosh, Saul Halfon, Katharine Legun, Sarah J. Martin

**Affiliations:** 1 University of Auckland, Auckland, Aotearoa New Zealand; 2 University of Munich, Munich, Germany; 3 University of California, Santa Cruz, USA; 4 University at Buffalo, Buffalo, USA; 5 National Farm Medicine Center at Marshfield Clinic Research Institute, Marshfield, USA; 6 Yale University, New Haven, USA; 7 Cornell University, Ithaca, USA; 8 Dartmouth College, Hanover, USA; 9 University of Rhode Island, Kingstown, USA; 10 MINES Paris - PSL / CNRS, Paris, France; 11 University of Münster, Münster, Germany; 12 York University, Toronto, Canada; 13 University of Ottawa, Ottawa, Canada; 14 Universitat Oberta de Catalunya, Barcelona, Spain; 15 University of California, Davis, USA; 16 Rowan University, Glassboro, NJ USA; 17 University of Montana, Missoula, USA; 18 University of Copenhagen, Copenhagen, Denmark; 19 Auburn University, Auburn, USA; 20 Arizona State University, Tempe, USA; 21 Virginia Tech, Blacksburg, USA; 22 Wageningen University, Wageningen, Netherlands; 23 Memorial University of Newfoundland and Labrador, St. John’s, Canada

**Keywords:** Intellectual communities; communities of practice; academic writing; science and technology studies (STS); agri-food

## Abstract

Scholarship flourishes in inclusive environments where open deliberations and generative feedback expand both individual and collective thinking. Many researchers, however, have limited access to such settings, and most conventional academic conferences fall short of promises to provide them. We have written this Field Report to share our methods for cultivating a vibrant intellectual community within the Science and Technology Studies Food and Agriculture Network (STSFAN). This is paired with insights from 21 network members on aspects that have allowed STSFAN to thrive, even amid a global pandemic. Our hope is that these insights will encourage others to cultivate their own intellectual communities, where they too can receive the support they need to deepen their scholarship and strengthen their intellectual relationships.

This is the most sustained intellectual community that I have found outside of my department. The level of discussion and interaction is extremely high and always generous. It remains a generative place for me to engage with like-minded scholars. I really appreciate the comfort in the group with sharing works in progress at various stages of completion, and the positive but critical spirit in which each work is approached. The meetings are often the highlight of my month. - STSFAN member

I absolutely look forward to the STSFAN discussion every month. I have rarely experienced so much positive feedback, sharing of thoughts and expertise in one hour before. I feel the community is extremely constructive. – STSFAN member

The community is engaged, with a purpose, I love the global scope and the “intergenerational” dimension. It is an intellectual space I did not have in my current or past departments, as I tend to be the only person doing food-ag stuff. - STSFAN member

## Where is intellectual community?

Scholarship flourishes in inclusive environments where open deliberations and generative feedback expand both individual and collective thinking. Many researchers, however, have limited access to such settings, and most conventional academic conferences fall short of promises to provide them. Scholars are familiar with this experience: we submit a proposal many months in advance, prepare a presentation, and travel to a conference where we may receive some questions about our scholarship but rarely the kind of constructive feedback we need to truly advance our work. Conferences also do little to intentionally cultivate ongoing professional relationships. These require regular opportunities for reciprocal interactions, which in turn foster trust and shared interest in mutual scholarly and career advancement.

While the COVID-19 pandemic and rising awareness about the climate impacts of international travel have increased opportunities to engage in online conferences, they have also left many scholars feeling both physically and intellectually isolated–sorely missing supportive exchanges with like-minded peers (Flaherty 2021; Lewy et al. 2022). This is all happening in the context of neoliberal universities that emphasize individual achievement over collective thinking and knowledge production (Mountz et al. 2015).

It is well established that collective learning is a situated, social process which can be facilitated within “communities of practice” (Lave and Wenger 1991). Such communities, often referred to as intellectual communities in academic spaces, may emerge informally, though are necessarily held together by shared interests and regular community interactions (Firpo et al. 2009; Wenger 1998). In competitive, under-resourced, and often crisis-ridden academic environments, scholars–and universities–often need to create such communities proactively, so as to provide opportunities for productive intellectual exchange around members’ works-in-progress.

We have written this Field Report to share our methods for cultivating a vibrant intellectual community within the Science and Technology Studies Food and Agriculture Network (STSFAN). This is paired with insights from 21 network members on aspects that have allowed STSFAN to thrive, even amid a global pandemic. Our hope is that these insights will encourage others to cultivate their own intellectual communities, where they too can receive the support they need to deepen their scholarship and to strengthen their intellectual relationships.

## Who is STSFAN?

STSFAN is the best place I have found to engage with colleagues at the intersection of food system research and critical interrogations of science and technology. - STSFAN member

A small group of us came up with the idea for STSFAN at the annual 2019 Society for the Social Studies of Science (4S) conference in New Orleans. We were all interested in building connections with scholars who, like ourselves, engaged in critical questions related to food, agriculture and technoscience. We thought an intellectual community would be particularly important given the interdisciplinary nature of our scholarship and the critical questions we are asking about agri-food futures-in-the-making.

To stay connected, we created an online communication channel and repository for shared information (Slack) (Firpo et al. 2009). We held our first online writing workshop soon after the onset of the global COVID-19 pandemic, which turned into regular and ongoing monthly workshops. Some members have been with the network from the start, while others have joined after hearing about the network from colleagues, conferences, mentors or academic advisors. The product of these efforts has been the emergence of an inclusive community culture, one which supports the generous exchange of ideas among scholars at various stages of their careers, and in different parts of the world.

**Fig. 1:**
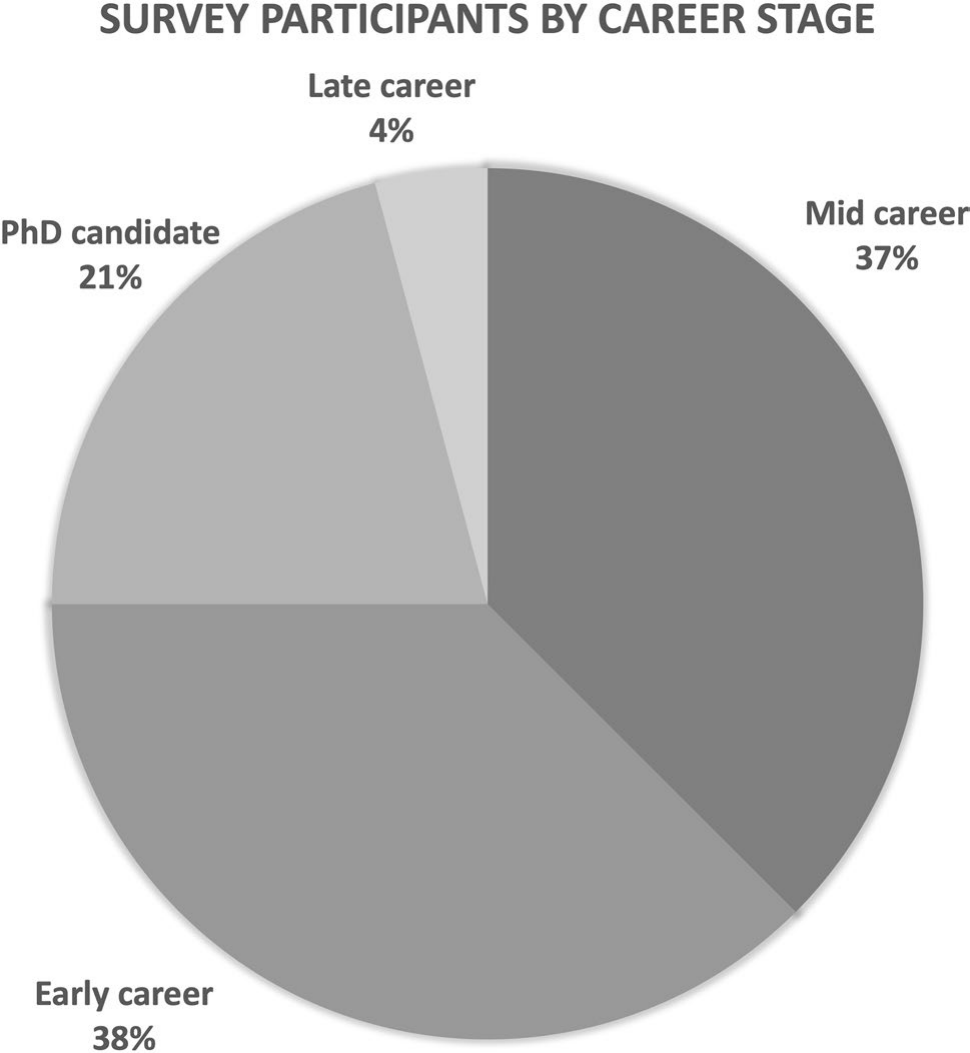
Career stages of the 21 STSFAN members whose feedback inform this Field Report

**Fig. 2:**
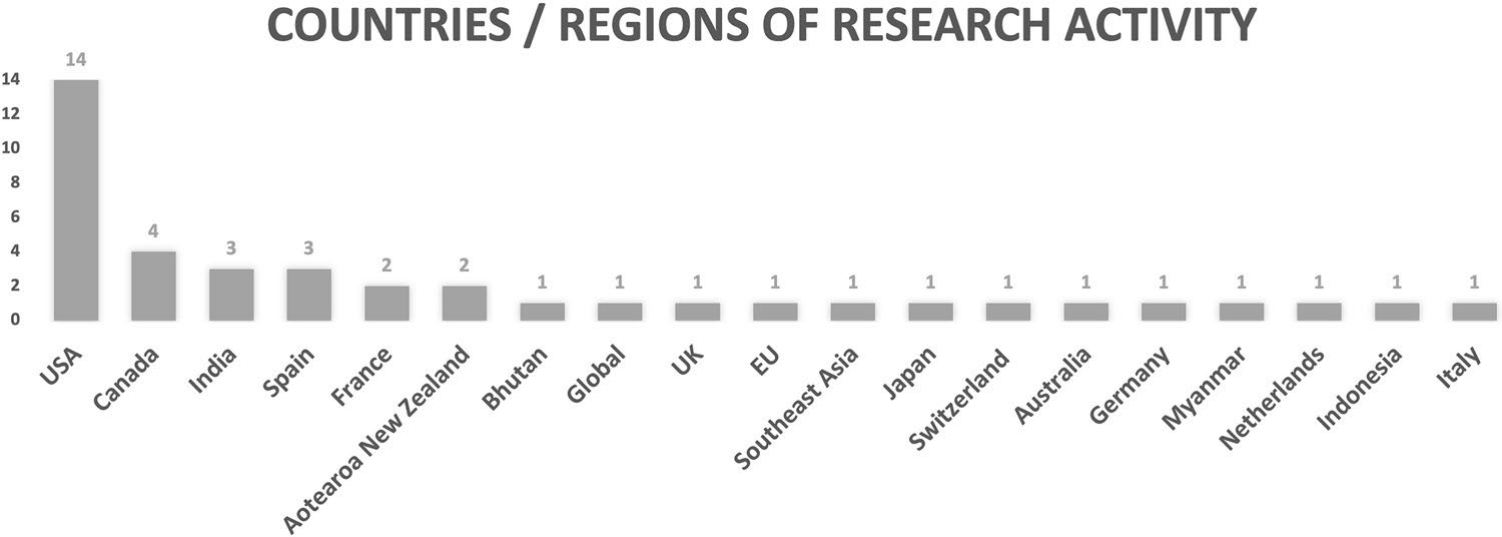
Countries or regions of research activity of the 21 STSFAN members whose feedback inform this Field Report

The 21 members whose insights inform this Field Report reflect the make-up of the larger STSFAN membership (approximately 150 people) in that they come from different countries, disciplines and career stages, and share an interest in science and technology studies (STS) approaches to agri-food. Figures 1 and 2 respectively illustrate the breakdown of these 21 members according to career stage and countries or regions where they conduct their research.

## What STSFAN does

### The monthly writing workshop

The STSFAN workshop model is generative, supportive and is a very good model for researchers to receive feedback during the writing process. It is teaching me how to read and review work in my field with a critical lens, asking what could make this work clearer and more impactful. - STSFAN member

STSFAN’s workshop style originated with the Yale Agrarian Studies Program and has been adopted and adapted in various other scholarly communities, such as the Berkeley Environmental Politics Colloquium where author Guthman first encountered it. In contrast to the traditional academic seminar where a scholar presents a paper and then defends it during a Q&A session, this workshop format prioritizes intellectual exchange aimed at improving the author’s paper (or chapter, or proposal), while also providing an opportunity for workshop participants to build professional relationships and engage in meaningful conversations about their shared topic of interest.

For STSFAN’s monthly workshops, authors share a draft (ideally not too polished) roughly a week in advance to participants, who in turn read the paper and prepare questions or comments to share with the group. (STSFAN participants sign up on an online spreadsheet to receive the paper by email). At the beginning of each session, the moderator gives the author five minutes to frame the work and mention specific concerns (e.g., fit for a particular journal, or specific questions they want workshop participants to think about). Then for the next 45 to 60 min the author remains silent while everyone else discusses the paper. Typically, participants pose a mix of conceptual and clarifying questions, suggest specific ways to strengthen the paper (i.e., through a shift in focus or organization, or by drawing on other literatures), but refrain from editorial comments. Ideally participants build on and debate each other’s comments (as opposed to taking turns listing off a number of unrelated comments). During this period, we ask workshop participants to refer to “the author” of the paper, rather than address the author as “you” or by name. This focus on the paper as a standalone artifact allows the author to be a temporary “fly on the wall”: they can listen to how others understand or react to their arguments without feeling defensive or compelled to respond, and can consider how they might frame those arguments more effectively. (In some settings authors take copious notes; since STSFAN meets virtually the session is recorded so that the author can focus on listening).

After the initial discussion, we invite the author to use the remaining time (approximately 30 min) however they want. During this final part of the workshop, participants speak to the author directly. The author might use this time to respond to questions raised, ask for further clarification about ways to improve the paper, or float new ideas for revision. We *strongly* discourage authors from taking a defensive position or responding to every comment. Instead, we encourage authors to ask clarifying questions to get as much feedback as possible. Our collective goal is for the author to walk away with ​​an idea of how readers understand their writing project to aid them in their writing process. Through this method, our workshops have supported the publication of a number of academic articles and book chapters (Biltekoff and Guthman 2022; Fairbairn et al. 2022; Guthman and Biltekoff 2022; Legun and Burch 2021; Reisman 2021; Schoot and Mather 2022). In addition, many authors leave our workshops with ideas on future writing projects, which sometimes emerge as collaborative writing projects with other STSFAN members (Broad and Biltekoff 2022; Guthman et al. 2022).

### Online workspace

I really appreciate the multifunctionality of the Slack channel (well, not the particular platform per say, but rather the opportunity for free-form interactions it enables). - STSFAN member

While the monthly workshops facilitate regular interactions which hold our intellectual community together (Wenger 1998), STSFAN members also connect via our online workspace. Monthly meetings may attract ten to thirty people, whereas the network’s approximately 150 members use our online workspace to share publications, information about jobs, conferences, calls for papers, and pedagogical materials. We also use the workspace to organize conference panels, engage in collaborative writing projects (including this Special Issue), and develop a network website.^1^ Thus, as a communication channel and an information clearinghouse, our online workspace cultivates relationships among STSFAN members who may not be able to attend every workshop but want to contribute to other collective endeavors.

### Administration

I want to recognize the administrative work and guidance that sustains the meetings, Slack etc. - STSFAN member

Despite often being neglected as invisible labor, we want to emphasize that cultivating intellectual community requires administrative work, for example, to set up an online communication platform, support new members, encourage members to share their work, etc. In STSFAN, we currently have dedicated members taking on this labor, though it could be distributed in various ways, for instance, divided by specific tasks or over specified periods of time.

##  Why STSFAN works

While a regular writing workshop and online workspace are two methods for establishing an intellectual community, a group’s vitality depends on the values which shape member interactions. In this section, we draw on insights shared by 21 STSFAN members which illustrate ten aspects that contribute to the network’s intellectual energy and inclusiveness.

### Accessibility and consistency

The STSFAN writing workshops have provided a consistent outlet for reading, thinking, and discussing cutting-edge scholarship. It’s had direct and indirect benefits for my own scholarship, and the collective impact of the gatherings has helped to move forward an evolving field of study. - STSFAN member

STSFAN’s monthly virtual meetings are fairly accessible to anyone with a laptop, internet access, and the willingness to meet once a month (though not always at an ideal hour, given that our members are spread across several time zones). Many members look forward to these monthly workshops, noting the value of their consistency (in occurrence and format).

### Shared curiosity

I have finally found a group of scholars who do research on food and agriculture in a way that I can relate to. - STSFAN member

STSFAN members’ shared interest in advancing STS approaches to food and agriculture allows for a depth of conversation–regardless of the topic of the particular work-in-progress. As one member put it, exchanges within STSFAN keep members “abreast of the current dialogues about food, agriculture, and science/technology.” At the same time, our discussions introduce scholars new to STS or agri-food to ongoing debates in these fields. Keeping up with relevant literature and debates is particularly important given the geographic expansion of the agri-food sector and the rapid pace at which innovations in food and agriculture emerge. Thus, network members appreciate opportunities for these ongoing exchanges which support them to engage in rigorous and timely scholarship in their respective locations, roles and projects.

### Mutual respect and inclusivity

This has been the most generous, welcoming, critical and regular intellectual group in all of my academic experience. There is a dire need in academic training to workshop what we write, especially for non-native English speakers, and this group really embodies that value. - STSFAN member

STSFAN members describe the monthly workshop as a space of mutual respect and inclusivity. Some women in the group, for example, see it as a space where they are less likely to be interrupted or mansplained than in other academic forums. In the words of one member: “I love that everyone brings such a spirit of mutual respect, generosity and gratitude to the process. I treasure our dynamic.”

### Flattened hierarchies

I cherish the care and open co-thinking spirit between junior and senior scholars, with its underlying ethos that academic knowledge production reflects dialogue (rather than individual minds). - STSFAN member

STSFAN includes scholars from all career stages (see Fig. 1), who put effort into creating a culture where contributions from each member are weighted equally. Early career scholars and graduate students are encouraged to critique the work of much more experienced scholars just as they would their peers. This creates a non-intimidating, non-competitive atmosphere where diverse contributions are welcomed with curiosity. Such experiences are not insignificant within an increasingly competitive academic environment, where students are either star-struck, or fear their contributions are not compelling or sharp enough to share with others.

### A focus on ideas

STSFAN feels like home. I do not always have to worry about being called out or abruptly attacked for a thought, because we focus on the paper–not on the person. - STSFAN member

The workshop’s scholarly discussions are valuable in two ways: first, they provide feedback at an early writing stage; second, they focus on intellectual ideas and not the person who wrote them. Bringing drafty papers and half-baked ideas make discussions more generative, and allows for argument clarification and the potential to move pieces of writing in unanticipated directions–something that is easier to do with draftier works-in-progress.

### Intellectual diversity

The feedback in an STSFAN workshop is as deep as a journal review, but it is more open, more diverse, more fun. - STSFAN member

The convergence of scholars across career stages, disciplines, geographic regions and institutions offers insights that many members could not find in other forums. Since members have different disciplinary backgrounds, workshops are an opportunity for participants to learn new theories or approaches, which can help them to better situate their own scholarship within the fields of STS and agri-food. As one member put it, “I doubt I am the only one making tentative forays into new intellectual crossroads with the support of this group.” These experiences are also particularly useful for students and early career academics, who can receive insights into what writing might look like at different stages of the academic career (e.g., translating a PhD chapter into an academic paper; writing a book proposal). It means workshops are also spaces to discuss more pragmatic dimensions of writing, such as style and journal choice.

### Intellectual generosity

The feedback is always very respectful and generous, while being honest and critical at the same time. - STSFAN member

Consistent, substantive, topically focused writing workshops form the core of STSFAN and in doing so demonstrate one of the group’s characteristic qualities: intellectual generosity. The group works because a critical mass of vibrant thinkers invest their time in supporting one another and are willing to be vulnerable in presenting early-stage writing. Perhaps most importantly, they do so while discarding the hierarchy, pretension, distrust, or theoretical boundary policing found in many academic spaces. This generosity and openness are supported by the structure of the workshops, initially silencing the author and building on ideas across comments rather than each individual enumerating suggestions, as well as the humble tone set by senior scholars leading by example.

### Accountability

I would describe the STSFAN writing workshops as a generous and generative space of scholarly accountability where we have focused discussions about works in progress. - STSFAN member

STSFAN members are aware they encourage a generous exchange of ideas within an academic environment riddled with warranted concerns about the theft of intellectual ideas. Thus, instead of circulating papers within the wider 150-person group, we promote accountability through an online sign-up sheet. Draft papers are only sent to STSFAN members who list their emails on this sheet, and the sheet itself remains a record of who has engaged with the work-in-progress. In that sense, our sign-up sheet serves as an organizational tool for workshops and as an accountability mechanism to ensure works-in-progress can be discussed in an open and generative way without fear that someone might scoop the ideas being shared.

### Initiative

There is space for many different activities and ideas, and it is truly run from the bottom. - STSFAN member

In addition to the cornerstone workshops, members have initiated additional collaborations, including writing collaborative papers, building a website, and organizing conference sessions or special issues. Such collaborative initiatives have not only led to new opportunities for members, but also expanded the wider group. For example, a series of STSFAN-organized sessions at the 4S, ASFS/AFHVS (Association for the Study of Food and Society and the Agriculture, Food and Human Values Society), and AAG (American Association of Geographers) conferences energized the group and grew its membership.

### Fun

So far, I have always gladly turned down other Friday night plans (21:30 − 23:30 CET) for the STSFAN meetings, which I take as a measure of how much I’ve enjoyed the meetings so far. - STSFAN member

Our members collectively refer to their engagements within STSFAN as fun. Many attribute this enjoyment to the genuine curiosity shared by other members, and the interesting conversations that emerge in both the workshops and online workspace. Sharing random, yet intriguing tidbits from the worlds of STS and agri-food (e.g., podcasts, news of luxury AgTech vessels, or Farms of the Future calendars from 1957!) and celebrating each other’s achievements provides a steady stream of uplifting engagement within the STSFAN community. Our members’ experiences highlight that maintaining a level of conviviality and enjoyment is key to the cohesion and success of a group. Put simply, fun should not be overlooked or understated when cultivating intellectual community.

## Conclusion

Communities are dynamic and vary widely based on the needs and goals of their constituent members. Thus, it is essential to acknowledge that cultivating intellectual community is an ongoing process that requires continuous reflection on what works, what doesn’t, and how to address limitations as they emerge. This is particularly important for a collective like STSFAN which values inclusivity. With this in mind, we have recently identified three limitations that we are working to address.

First, while we consider ourselves a global community, we recognize a North American bias, not only in that many members are based in the United States and Canada, but also in the chosen time for our workshops. Second, STSFAN converses in English, which may exclude scholars that feel less confident with engaging in complex intellectual conversations in that language. Third, access to STSFAN workshops are limited to people with well-functioning computer and internet infrastructures.

Moving forward, we plan to address these limitations by conducting regular internal surveys to find the best time slot for the majority of active members and working to broaden our member base. This will require that we more clearly articulate our shared practices, values and commitments to mutual respect and inclusivity–which we are doing in this Field Report and on our website. With these points in mind, we look forward to further cultivating STSFAN, and hope to soon welcome you as a new member. Alternatively, if our experience has inspired you to cultivate your own intellectual community, we would love to hear how it goes. For more information on STSFAN, please visit https://stsfanetwork.wixsite.com/stsfan.

## Footnotes

1 https://stsfanetwork.wixsite.com/stsfan.
